# The development of a stochastic mathematical model of Alzheimer’s disease to help improve the design of clinical trials of potential treatments

**DOI:** 10.1371/journal.pone.0190615

**Published:** 2018-01-29

**Authors:** Christoforos Hadjichrysanthou, Alison K. Ower, Frank de Wolf, Roy M. Anderson, for the Alzheimer's Disease Neuroimaging Initiative

**Affiliations:** 1 Department of Infectious Disease Epidemiology, School of Public Health, Imperial College London, London, United Kingdom; 2 Janssen Prevention Center, Leiden, The Netherlands; Nathan S Kline Institute, UNITED STATES

## Abstract

Alzheimer’s disease (AD) is a neurodegenerative disorder characterised by a slow progressive deterioration of cognitive capacity. Drugs are urgently needed for the treatment of AD and unfortunately almost all clinical trials of AD drug candidates have failed or been discontinued to date. Mathematical, computational and statistical tools can be employed in the construction of clinical trial simulators to assist in the improvement of trial design and enhance the chances of success of potential new therapies. Based on the analysis of a set of clinical data provided by the Alzheimer's Disease Neuroimaging Initiative (ADNI) we developed a simple stochastic mathematical model to simulate the development and progression of Alzheimer’s in a longitudinal cohort study. We show how this modelling framework could be used to assess the effect and the chances of success of hypothetical treatments that are administered at different stages and delay disease development. We demonstrate that the detection of the true efficacy of an AD treatment can be very challenging, even if the treatment is highly effective. An important reason behind the inability to detect signals of efficacy in a clinical trial in this therapy area could be the high between- and within-individual variability in the measurement of diagnostic markers and endpoints, which consequently results in the misdiagnosis of an individual’s disease state.

## Introduction

Alzheimer’s disease (AD) is a neurodegenerative disorder which is characterised by progressive accumulation of amyloid-beta (Aβ) peptides, forming senile plaques in the brain, and neurofibrillary tangles of hyperphosphorylated tau, followed by progressive neuronal and synaptic loss. It has long preclinical and prodromal stages and affects memory, thinking, behaviour, daily functional abilities, and emotion. Alzheimer’s disease may account for approximately 60–80% of dementia cases [1]. The worldwide prevalence of dementia was estimated to be over 46 million people, with studies suggesting that the number of cases is rising exponentially with age and that the above estimate will almost triple by 2050 [2–4]. Apart from the health and social problems, Alzheimer’s, and dementias in general, have huge economic impact, with the estimated worldwide cost of dementia approaching USD 818 billion [4–6]. Thus, drugs are urgently needed to improve therapies of AD and dementia.

There are two competing models proposed to explain the hallmarks of AD: the amyloid hypothesis (the neuron-centric model) [7, 8] and the Inverse Warburg hypothesis (the neuron-astrocytic model) [9, 10]. The neuron-centric model suggests that a mutation in the nuclear genome induce overproduction of Aβ and tau that become toxic to neurons. The neuron-astrocytic model contends that the progression of AD is triggered by defects in the normal energy transduction process, a condition induced by mitochondrial dysregulation. Although some clinical trials of metabolic interventions have shown promising results for the improvement of cognitive performance [11, 12], clinical trials of disease-modifying treatments have consistently failed. A recent study reviewed 413 clinical trials (124 Phase 1 trials, 206 Phase 2 trials, and 83 Phase 3 trials) of AD drug candidates that have been conducted between 2002 and 2012 [13]. The study reported that 99.6% of the trials failed or had been discontinued. No cure or other treatments that prevent, halt or reverse the underlying pathology of established AD have been approved as yet. Available treatments, such as the cholinesterase inhibitors tacrine (1993), donepezil (1996), rivastigmine (1998) and galantamine (2001), are approved for mild to moderate AD and can provide only temporary improvement in cognitive and behavioural symptoms, and at best a temporary impact on the progression of the underlying pathology of the disease [14].

The failure rate of AD clinical trials is far higher than that of trials in other therapy areas, and information on the reasons of trial failure or discontinuation is limited [13, 15]. Some of the reasons of high trial failure include the poorly understood nature of AD pathogenesis and progression and poor trial design. For example, the wrong choice of clinical endpoints and markers used as clinical trial entry criteria, as well as the selection of unsuitable individuals for enrolment in the trial. The retention of participants due to the long duration of the AD trials is also an important problem to overcome in the design of successful clinical trials in the AD area [15]. The high failure rate of clinical trials can also be attributed to the inability to detect signals of efficacy, even if the treatment is safe and effective. This could be due to the administration of the drug at a relatively late stage of the disease, at which time the condition of the patient may be irreversible, or due to the high variability in the measurements of different biochemical and cognitive markers that are used as diagnostic markers and endpoints.

Mathematical modelling of AD progression and the development of clinical trial simulations are essential tools for exploring the reasons why a clinical trial can fail and for the improvement of the design of clinical trials. The poorly understood nature of AD pathogenesis and progression limits the possibilities of developing robust mechanistic models for the accurate prediction of disease progression. However, a few attempts have been made to develop models that describe the development of AD at the molecular level (e.g. [16–20]). Published mathematical models also include semi-mechanistic models developed to describe longitudinal changes in measures of cognition, as assessed by errors in various cognitive tests designed for the assessments of the cognitive capabilities of the patients, such as the Modified Mini-Mental State Examination (MMSE) and the Alzheimer’s Disease Assessment Scale (ADAS-cog) [21–25]. In general, published research on the mathematical modelling of AD has mainly focused on: (1) the study of the epidemiology of AD (e.g. [26, 27]); (2) the investigation of the progression of the disease based on changes in the cognitive status (e.g. [21–25]); (3) on defining the order of the various biomarkers of the disease in predicting progression (e.g. [28–40]), and (4) on the description of the pathogenesis and evolution of the disease at the molecular level (e.g. [16–20]). Most of the models developed so far are stochastic in nature [41]. Such models permit variability in the model parameters and chance elements in AD disease progression.

In this study we develop and analyse a simple stochastic model to predict AD progression in a clinical trial. In particular, we define a discrete-time Markov model to describe the movement of individuals through a finite sequence of distinct health and disease states over time (distinct time points at which the state of the individuals is assessed). Markov models are ideal for the modelling of AD, as this disorder can be described as a multi-state disease process, and due to the observational nature of studies of AD in which the condition of individuals is assessed at periodic visits. These are also useful tools for the estimation of the impact of risk factors on the transitions between the states and can easily include reversible states that have been observed in many AD cohort studies. This modelling framework can contribute to the improved design of future clinical trials by providing insights into what to measure, the importance of measurement error in determining efficacy, sample size selection, duration of the trial and other important factors such as demographic characteristics of the sample. Markov models have also been purposed in previous studies for the estimation of the probabilities of transitioning between different stages of AD severity, and the investigation of potential covariates that can influence these probabilities [14, 41–51]. In this study, we use this framework to develop tools for the assessment and evaluation of the impact of receiving a hypothetical preventative treatment of varying efficacy, at different stages of disease progression, and illustrate the potential problems detecting significant differences between treated and untreated individuals. The specific action of the treatments in the model is irrelevant; the treatments are assumed to reduce the probability of transitioning to a more severe stage of the disease, which consequently slows the development and progression of the disease.

The transition probability distributions of the Markov model we describe are estimated using a particular longitudinal cohort dataset provided by the Alzheimer's Disease Neuroimaging Initiative (ADNI, adni.loni.usc.edu).

## Material and methods

### The model

The development and progression of AD in a clinical trial was assumed to be a stochastic process. In particular, we developed a discrete-time Markov chain in a random environment defined on a finite state space, with the property that for all states the probability of moving from the current state to the next state is independent of the past. Individuals were classified according to their disease status. In particular, based on a set of criteria individuals were categorised as Cognitively Normal (CN), with Mild Cognitive Impairment (MCI) and with AD. The criteria are based on the level or presence of a number of markers, including but not limited to memory complaints, logical memory II subscale, MMSE, CDR, and NINCDS/ADRDA criteria for probable AD [52]. The states are distinct and do not overlap.

**Fig 1:**
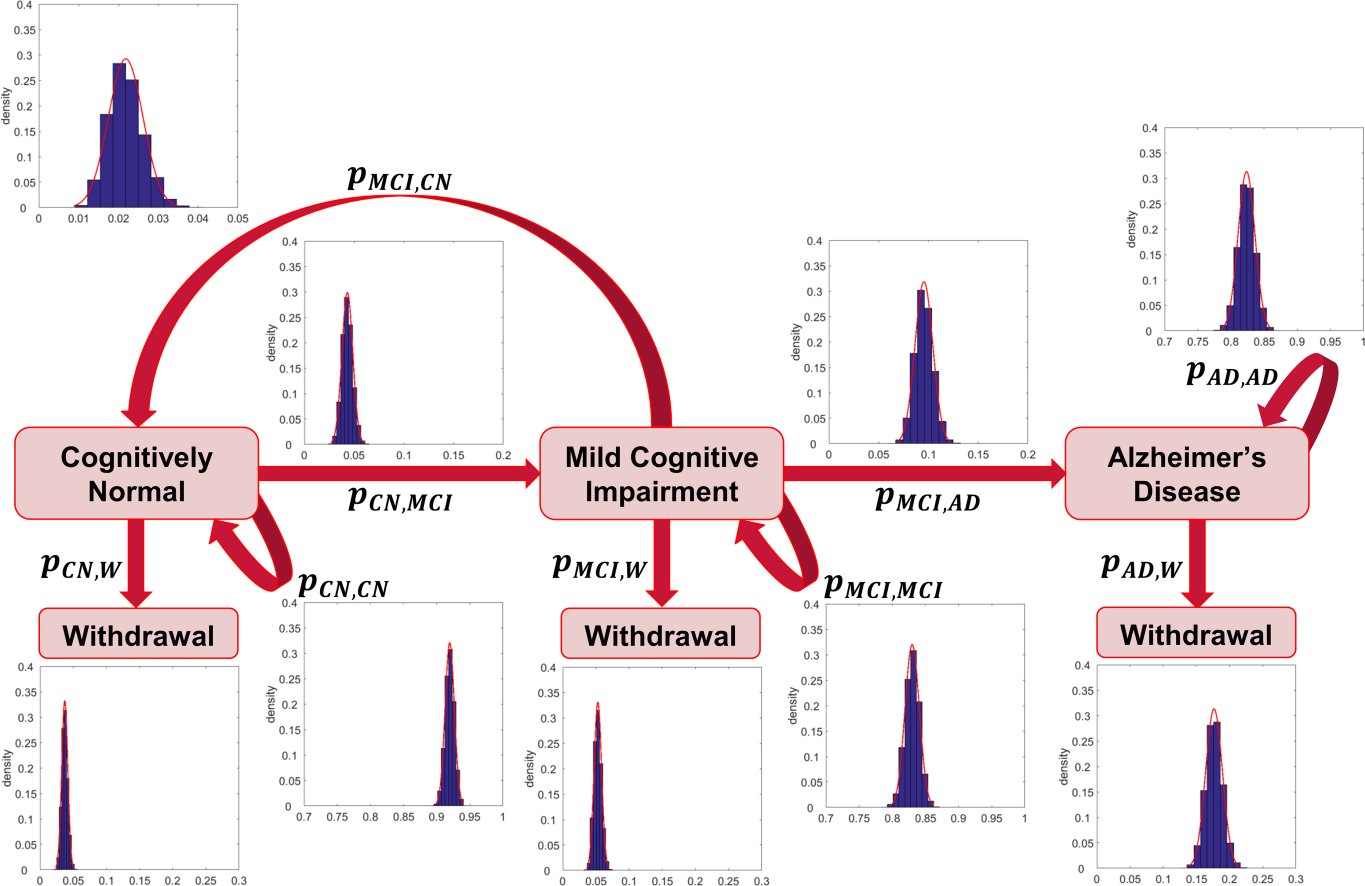
Flow diagram of possible transitions between the different states of the Markov model. Individuals can be in one of the following states: Cognitively Normal, Mild Cognitive Impairment, Alzheimer’s Disease and Withdrawal. Arrows indicate possible transitions between the states. *p*_*A*,*B*_ represents the probability of moving from state A to state B. The estimated distributions of the average one-year transition probabilities from the ADNI cohort dataset are presented.

In each (discrete) time step of the process, individuals can either withdraw from the study, for example due to death or personal reasons, remain at the same state or move to a different state with some probability (transition probability). Based on the information obtained from the ADNI dataset, transitions are allowed between the states CN and MCI in both directions (i.e. recovery to normal cognition is also allowed) and from the MCI to the AD state. Back-transitions may be due to either measurement error or a genuine ability in some patients to show cognitive improvement (e.g. due to cognitive reserve). In the ADNI dataset, a small number of transitions from AD back to MCI is also observed (a total of 17 transitions in yearly assessments across the ten-year cohort). Some reverse transitions from AD to MCI, as well as from MCI back to CN, have also been recorded in other longitudinal studies [50, 53–56]. However, due to the progressive nature of AD and the fact that AD diagnosis cannot be confirmed until an autopsy is performed, in the theoretical model that we developed we do not allow transitions from AD back to MCI state, i.e. once patients reach the AD state their condition cannot improve. Even in the presence of candidate treatments that could potentially reverse the pathology at a stage of AD (e.g. [57, 58]), there is no sufficient evidence that the cognitive functions and clinical outcome of AD individuals can be improved. Due to the stochastic nature of the model, allowing both an MCI to CN and an AD to MCI transition would even mean that there will always be a chance of patients becoming fully recovered from AD, i.e. returning to a cognitively normal state, an outcome that cannot be supported at present. However, it should be noted that incorporating an AD to MCI transition in the model would have a negligible effect on the output due to the small chance of this transition happening. A small number of patients in ADNI also transitioned from CN directly to AD (3 patients in yearly assessments across the ten-year cohort). These transitions have also been attributed to potential misclassifications of patients and not been incorporated into the model (see [59]). A schematic diagram of the model developed is presented in Fig 1.

### Transition probabilities

For the estimation of the transition probabilities between the different states we followed a piecewise approach. We divided the observation period into one-year intervals and calculated the transition probabilities in each of these intervals (in ADNI, data exists for a total of 10 years). The probabilities of transitioning between states were estimated independently for each time interval, and it was assumed that the process is temporarily homogeneous within these intervals, i.e. the transition probabilities were assumed constant within each of the time intervals.

Let $p_{S_{i},S_{j}}(t)$ be the transition probability to state S_*j*_ at time *t* given that at time *t* − 1 the state is S_*i*_, where *i*, *j* ∈{1, 2,…, *K*} and S_*i*_, S_*j*_ ∈*S*. *S* is the state space of the Markov Chain and *K* is the number of states. The maximum likelihood estimate of the transition probability $p_{S_{i},S_{j}}(t)$ is given by

$$p_{S_{i},S_{j}}(t)=\frac{\tau_{S_{i},S_{j}}(t)}{\sum_{l=1}^{K}\tau_{S_{i},S_{l}}(t)},$$ (1)

where $\tau_{S_{i},S_{j}}(t)`$ is the number of transitions from S_*i*_ (at time *t* – 1) to S_*j*_ (at time *t*). $\overset{K}{\underset{j=1}{\sum }}p_{S_{i},S_{j}}=1$ for all *i*.

In order to assess the uncertainty of estimates of each transition probability we used the bootstrap method [60, 61]. With this method, a number of possible cohort datasets are generated to develop a range of possible transition matrices. Specifically, for each time interval new sets of transitions are generated by sampling with replacement from the original set of transitions (the number of transitions in the generated samples is the same as the total number of transitions in the observational study). Combining all the bootstrapped transition matrices we derive an approximation of the sampling distribution [61]. To smooth the distributions of the transition probabilities we fitted a Normal-distribution using the maximum likelihood estimation method.

The accurate estimation of transition probabilities is particularly challenging, mainly due to the heterogeneity of the data, the infrequent observations on the same patient, and missing data points [62]. It should be noted that although the total number of individuals in the study that we used is relatively large (1624 individuals, see subsection ‘Dataset used’), this number decreases significantly over follow-up, either because individuals die or are lost to follow-up. It is also important to note that our analysis uses follow-up time as opposed to chronological time, such that those subjects recruited later (into the newer ADNI-GO or ADNI-2 cohorts, see subsection ‘Dataset used’) will have inherently less follow-up time than those enrolled initially into the study (ADNI-1). Thus, we naturally lose depth in the number of subjects at later follow-up times due to recruitment date.

### Stochastic simulation

Within the modelling framework described, we numerically simulated the time progression of the disease in a sample of individuals in a clinical trial. As an example, in this preliminary study the development and progression of the disease was simulated in a general population of 1000 individuals for duration of up to 10 years. The trial duration was discretised into uniform steps. The time-step was defined to be one-month. At the beginning of each simulation, a value for each transition probability was assigned to each individual in the trial. The value was drawn at random from the respective distribution of the average one-year transition probability. In each time-step in the simulation (same for all individuals), each individual moved independently from one state to another state with the defined transition probability. The state of each individual was updated synchronously. For each numerical simulation we performed 10^4^ realisations. We focused on the proportion of AD cases over time. We are mainly interested in the effect of potential preventative treatments, before the occurrence of AD clinical symptoms. For this reason, we considered optimistic treatments focusing on the case where at the beginning of the process all individuals are cognitively normal. The other extreme where all individuals are initially at the MCI state was also considered. It should be noted that, given the assumption that AD is a progressive disorder, and thus AD is an irreversible state, including a number of AD individuals in the initial population would have the same effect as removing these individuals completely from the process.

The simulator was developed in MATLAB (R2017b). The computer code can be obtained from the authors upon request.

### Impact of interventions

Interventions were applied at different time points of the process. In our analysis we focused on treatments that, irrespective of their specific action, reduce the probability of transitioning from state X to a more severe state Y by a proportion *E*_*X*,*Y*_ ∈ (0,1). Despite the nature of the dataset we used, we focused on preventative treatments that are administered to a sample of cognitively normal individuals in order to reduce the probability of transitioning to MCI and later from MCI to AD. The same treatments were also assessed in a sample consisting of only MCI individuals. We also simulated clinical trials to evaluate the effect of treatments that are administered to a sample of only cognitively normal individuals, but the treatment becomes effective only at the MCI state and reduces the probability of developing AD. The effect of a treatment that reduces the probability of transitioning from CN to MCI has a negligible effect when administered to individuals that have already reached the MCI state. To investigate the impact of treatments that might not be effective beyond a point, as may happen, for example, due to severe neuronal degeneration, we also considered treatments that are administered before the MCI onset to reduce only the probability of developing MCI, i.e. they are no longer effective beyond the MCI state. Indeed, the model also allows the consideration of the effectiveness of treatments that might vary with time, but we did not consider this scenario in the current study. All the treatment effects were assumed to be homogeneous between individuals. We focused on the ideal scenario where treatments have an immediate effect, but we also considered cases where there are time delays, which may refer either to delays in the administration of treatments that are immediately effective or delays before treatments that are administered at the beginning of the trial start to be effective. Indeed, if a clinical trial involving ideal treatments which have an immediate effect and their effectiveness is not reduced over time is unsuccessful, we expect that clinical trials of less effective treatments (example, clinical trials of treatments which have delayed and/or time-decaying effects) will also be unsuccessful. We assessed the impact of interventions on the proportion of AD cases over time. The effect of a treatment at a specific time point is defined as the mean difference between the proportion of AD cases in the untreated and treated groups. Other potential endpoints that could be studied using the model include the incidence proportion of AD cases, the expected time spent in a disease state, and the expected time and probability to reach a specific state from any other state. Information for each of these endpoints is generated in the simulation.

For every simulation output we present the predicted value of the proportion of AD cases at the end of a 5- and a 10-year trial under different intervention scenarios. We calculated the 95% credible interval of the distribution of the proportion of AD cases and the standard deviation. Although outside the scope of the current study, it should be noted that the results of such simulation exercises could also be used as prior information required for sample size calculations before the conduct of a clinical trial. For every case, we performed a hypothesis test for the equality of two binomial proportions, the proportions of AD cases in the treated and untreated groups, i.e. we tested whether the difference of the two proportions is equal to 0. A two-tailed test was performed and statistical significance was calculated using a 95% confidence level.

### Dataset used

For the development of the Markov model and the estimation of the model parameters we used data obtained from the Alzheimer’s Disease Neuroimaging Initiative (ADNI) database (adni.loni.usc.edu, last downloaded 2016/10/31). The ADNI was launched in 2003 as a public-private partnership, led by Principal Investigator Michael W. Weiner, MD. The primary goal of ADNI has been to test whether serial magnetic resonance imaging (MRI), positron emission tomography (PET), other biological markers, and clinical and neuropsychological assessment can be combined to measure the progression of mild cognitive impairment (MCI) and early Alzheimer’s disease (AD). For up-to-date information, see www.adni-info.org.

The dataset used is a longitudinal observational study consisting of 1737 individuals (from the ADNI-1, ADNI-GO, and ADNI-2 protocols), 106 of which were categorised as ‘Significant Memory Concern’ (SMC) at baseline and excluded from our analysis. In addition, there were 7 individuals that had only a screening visit and they were also excluded. Therefore, in the present study, we analysed data from 1624 participants. The characteristics of the individuals in the dataset considered are presented in Supporting Information, S1 Table. The maximum duration for which a participant in the study has been followed up is 10 years. It should be noted that in the original dataset, individuals in the MCI state are further classified as ‘Early MCI’ (EMCI) and ‘Late MCI’ (LMCI). However, in this analysis we combined the two MCI subtypes.

The status of individuals was assessed only at the distinct time points considered in the model. The number of transitions between the states of the Markov model at each time point is presented in Supporting Information, S2 Table.

## Results

**Fig 2:**
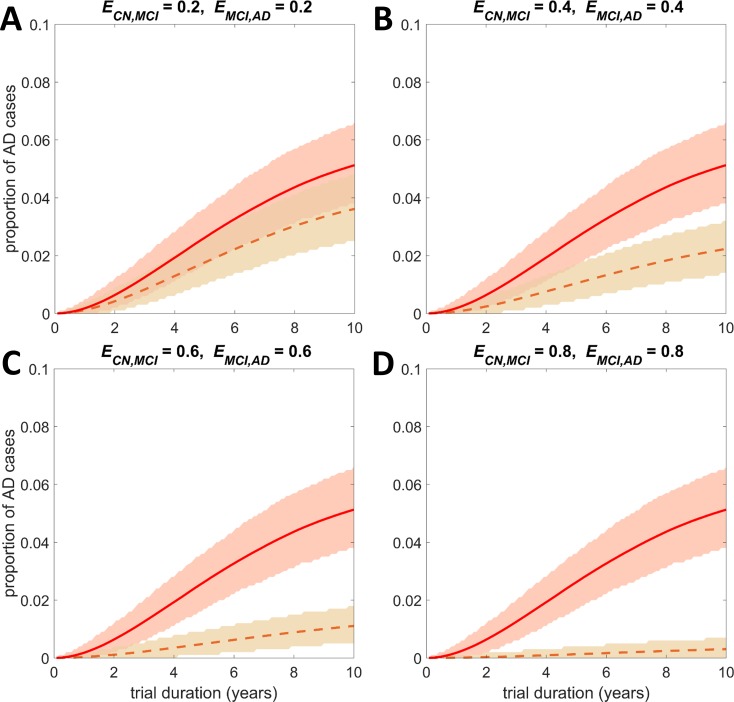
Output of stochastic simulations: The expected proportion of AD cases over time and the effect of treatments that reduce the transition probabilities *p*_*CN*,*MCI*_ and *p*_*MCI*,*AD*_ in a sample of CN individuals. The expected proportion of AD cases over time in the untreated group is presented by the solid line. The expected proportions after the administration of potential treatments that reduce the transition probabilities *p*_*CN*,*MCI*_ and *p*_*MCI*,*AD*_ by a proportion *E*_*CN*,*MCI*_ and *E*_*MCI*,*AD*_, respectively, are presented by the dashed lines. (A) *E*_*CN*,*MCI*_ = 0.2, *E*_*MCI*,*AD*_ = 0.2, (B) *E*_*CN*,*MCI*_ = 0.4, *E*_*MCI*,*AD*_ = 0.4, (C) *E*_*CN*,*MCI*_ = 0.6, *E*_*MCI*,*AD*_ = 0.6, (D) *E*_*CN*,*MCI*_ = 0.8, *E*_*MCI*,*AD*_ = 0.8. The value of *E* can be interpreted as the efficacy of the candidate treatment, with *E* = 0 implying no efficacy and *E* = 1 a 100% efficacy which stops progression. The ‘shaded area’ represents the 95% credible interval of the distribution of the proportion of AD cases. At the beginning of the trial all individuals are at the CN state. The population size in each group is *N* = 1000.

**Fig 3:**
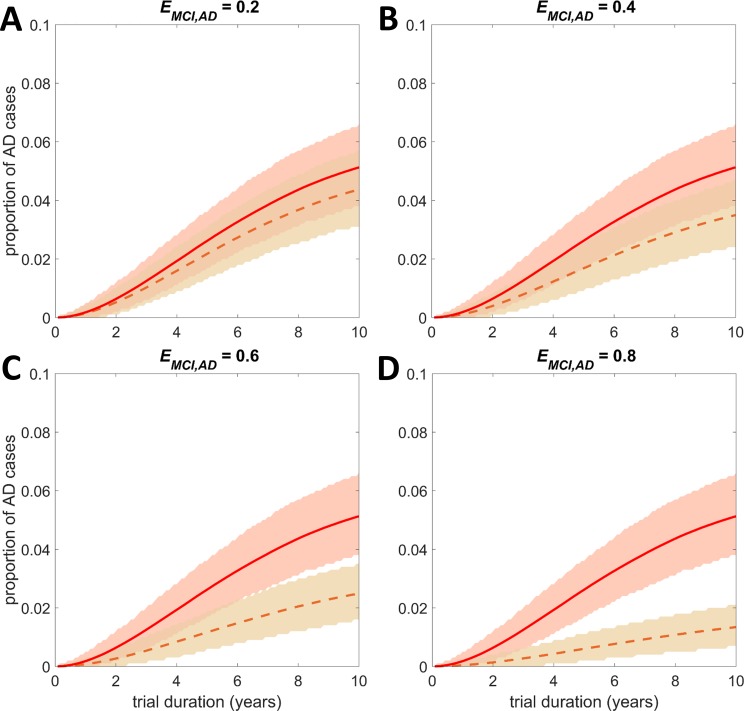
Output of stochastic simulations: The expected proportion of AD cases over time and the effect of treatments that reduce the transition probability *p*_*MCI*,*AD*_ in a sample of CN individuals. The expected proportion of AD cases over time in the untreated group is presented by the solid line. The expected proportions after the administration of potential treatments that reduce the transition probability *p*_*MCI*,*AD*_ by a proportion *E*_*MCI*,*AD*_ are presented by the dashed lines. (A) *E*_*MCI*,*AD*_ = 0.2, (B) *E*_*MCI*,*AD*_ = 0.4, (C) *E*_*MCI*,*AD*_ = 0.6, (D) *E*_*MCI*,*AD*_ = 0.8. The value of *E* can be interpreted as the efficacy of the candidate treatment, with *E* = 0 implying no efficacy and *E* = 1 a 100% efficacy which stops progression. The ‘shaded area’ represents the 95% credible interval of the distribution of the proportion of AD cases. At the beginning of the trial all individuals are at the CN state. The population size in each group is *N* = 1000.

Fig 1 illustrates the distributions of the average one-year transition probabilities (for the parameters of the fitted Normal distributions see Table 1). Fig 2, Fig 3 and S1 Fig show the output of stochastic simulations (expected proportion of AD cases over time in a general population of 1000 individuals) in the untreated and treated groups under different intervention scenarios. In all the examples presented in these figures we assumed that individuals are initially at the CN state. S2 Fig shows the output of simulations for the case where treatments reduce the probability of transitioning from CN to MCI and from MCI to AD, and all individuals are initially at the MCI state. In each of the above figures, the uncertainty in the output of the stochastic simulations in both the treated and untreated groups is also illustrated. The expected values of the proportion of AD cases at the end of a 5- and a 10-year simulated trial where all individuals are initially cognitively normal are provided in S3 Table. In S4 Table we present the results obtained at the end of a 5-year simulated trial for the cases where all individuals are initially at the MCI state. For each case, the 95% credible interval and the standard deviation are also presented. In S5 and S6 Tables we present the *p*-values of the hypothesis tests for the equality of the proportions of AD cases in the untreated and treated groups under the different intervention scenarios that have been investigated.

**Table 1:** Fitted normal distributions for the average one-year transition probabilities between the states of the Markov model. SD: standard deviation.

Average one-year transition probabilities	CN→MCI	MCI→CN	MCI→AD
	mean = 0.0432, SD = 0.0055	mean = 0.0218, SD = 0.0044	mean = 0.0954, SD = 0.0090
	CN→Withdrawal	MCI→Withdrawal	AD→Withdrawal
	mean = 0.0372, SD = 0.0046	mean = 0.0528, SD = 0.0057	mean = 0.1765, SD = 0.0127
	CN→CN	MCI→MCI	AD→AD
	mean = 0.9197, SD = 0.0070	mean = 0.8300, SD = 0.0108	mean = 0.8235, SD = 0.0127

It is observed that although treatments might be effective in reducing the chance of developing AD, due to the high uncertainty it might be difficult to assess their real effectiveness in a clinical trial. In particular, it is remarkable that in the sample of cognitively normal individuals alone, the simulation suggests that in a clinical trial of duration of five years, it is likely that the true efficacy of a treatment will not be detected even if the treatment has moderate efficacy (Fig 2, S3 and S5 Tables). As expected, if all the participants are initially at the CN state, a difference between the untreated and the treated group becomes even more challenging if the treatment is effective only when individuals are at the MCI state (Fig 3, S3 and S5 Tables). In particular, in a sample of cognitively normal individuals, treatments that reduce both the transition probability of moving from CN to MCI and the probability of moving from MCI to AD are the most effective, followed by treatments that reduce only the probability of transitioning from CN to MCI and then the treatments that reduce the probability of developing AD from the MCI state. Increasing the duration of the trial and the population size improves the chances of clinical success in all cases (see for example S3 and S5 Tables). The efficacy of a treatment that reduces the probability of transitioning to more severe states may be more likely to be detected in a trial where all individuals are initially at the MCI state (S2 Fig, S4 and S6 Tables). However, the proportion of AD cases at the end of such a trial is much higher. As mentioned earlier, in the analysis performed we showed that the effect of a treatment that reduces the probability of moving from CN to MCI state is negligible when it is administered to MCI individuals.

**Fig 4:**
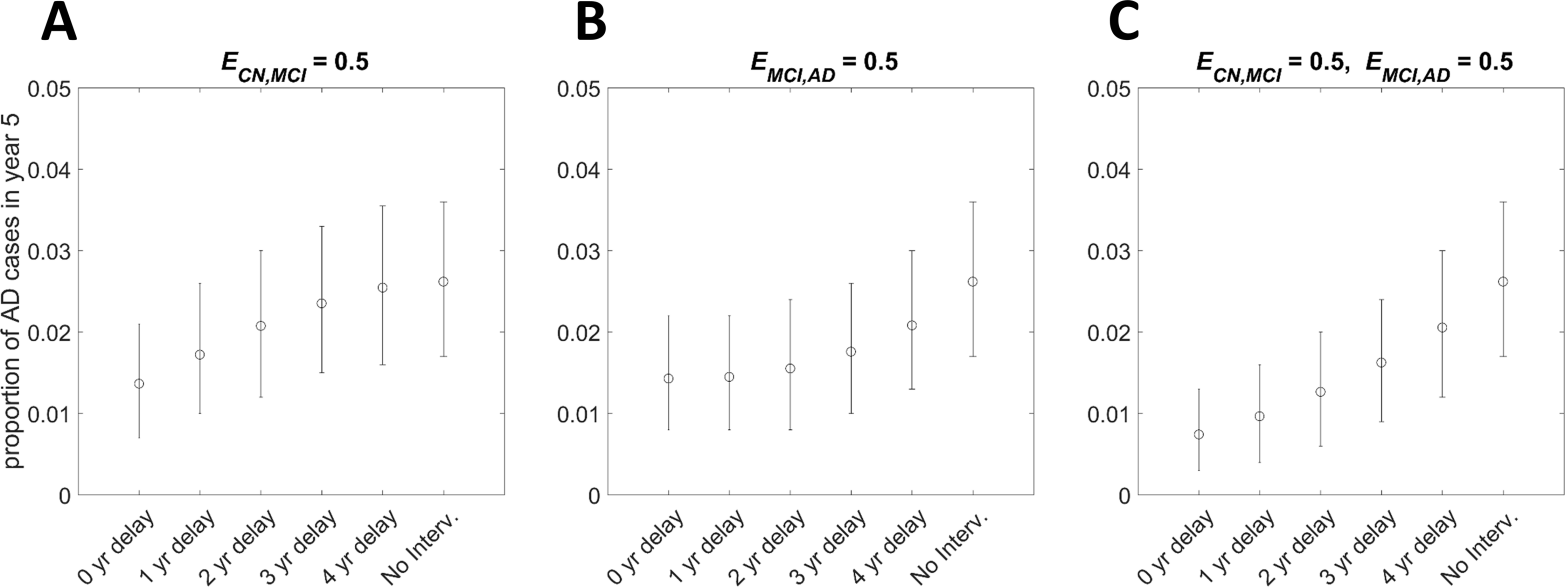
The expected proportion of AD cases at the end of a 5-year trial and the impact of time delays in the activation of various treatments in a sample of CN individuals. Treatments that reduce the transition probabilities (A) *p*_*CN*,*MCI*_ only, (B) *p*_*MCI*,*AD*_ only, (C) *p*_*CN*,*MCI*_ and *p*_*MCI*,*AD*_, by a proportion 0.5 are effective after some time delay. Circles represent the expected proportion of AD cases at the end of a 5-year trial and the error bars the 95% credible interval. At the beginning of the trial all individuals are at the CN state. The population size in each group is *N* = 1000.

Fig 4 illustrates the impact of time delays, before the treatment starts to be effective, on the proportion of AD cases at the end of a 5-year simulated trial. The treatment is administered to reduce the probabilities of transitioning to more severe states (*p*_*CN*,*MCI*_ only, *p*_*MCI*,*AD*_ only, *p*_*CN*,*MCI*_ and *p*_*MCI*,*AD*_). The impact of such delays on the proportion of AD cases at the end of a 10-year trial in the same scenario is illustrated in S3 Fig. In both Fig 4 and S3 Fig all individuals are initially cognitively normal. S4 Fig shows the impact of time delays on the effect of a treatment that reduces the transition probabilities from CN to MCI and from MCI to AD when the initial population consists of only MCI individuals. Fig 4, as well as S3 and S4 Figs, can also be thought of as the case where there is a period of delay before the administration of treatments that are immediately effective. Such delays influence the population distribution at the time of treatment administration; the initial number of CN individuals decreases and the number of MCI and AD individuals increases. It is shown that treatments administered to CN individuals are likely to be more effective in slowing AD progression if they are administered at the beginning of the study (*t* = 0) to reduce the probabilities of moving to MCI and later to AD state (see Fig 4, S3 and S5 Tables). However, the impact of time delays is more pronounced in the cases where treatments given to CN individuals reduce their chance of transitioning to the MCI state (but see S4 Fig). This is partly due to the fact that delays in the ‘activation’ of the treatment result in a reduction in the number of CN individuals who can benefit from the treatment, as a consequence of disease development between the initiation of the study and treatment activation. Consequently, after some delay, treatments administered to CN individuals to reduce the probability of developing AD from the MCI state may be more effective than treatments administered to CN individuals to reduce only the probability of moving to the MCI state. Hence, in a clinical trial the specific time of intervention is crucial.

## Discussion

Using the ADNI dataset, we developed a basic framework for the simulation of the development and progression of AD in a clinical trial to facilitate the assessment of the effect of potential candidate treatments. Treatments slow the transition to a more severe state, and thus disease progression. This reduction in the progression rate of the disease can be associated with a significant decrease in the incidence of AD, increase in the duration of individuals’ independence and the time until institutionalisation, and concomitantly a significant decrease in the costs of care. It is interesting to note that if such interventions could delay both disease onset and disease progression by even one year, the global prevalence of AD could decrease by almost 9.2 million, by the year 2050 [3].

It has been demonstrated that the chance of identifying the true treatment effect may be challenging due to the high uncertainty in the clinical trial outcome, even if the treatment has high efficacy. This can be one of the dominant causes of trial failure of AD treatments. The high uncertainty, which can be illustrated by the output of the simulations, can be attributed partly to the sparsity and heterogeneity of the available data, and the high variance in the measurements of markers that have been used for the classification of individuals in the different disease states. The simple criteria used for the classification of individuals, such as the performance on cognitive tests, and potential associated measurement error, may influence the correct diagnosis and could be an important reason of the presence of the high variability. Other reasons that can introduce variability in the measurements, both within and between individuals, and influence the diagnosis of the actual clinical stage include underlying medical conditions such as the presence of depression or psychosis, inter-clinician differences in application of diagnostic criteria, and resistance to cognitive decline due to cognitive reserve [42, 47, 63–65]. As some of these factors can significantly contribute to variance in the model parameters representing disease progression, they should potentially be considered as covariates in extended models.

It should be noted that, due to the nature of the ADNI data used in this study, our outputs may not reflect the progression and impact of the disease in the general population, and indeed the output in prevention clinical trials considered here. Participants in this dataset have been selected to mimic that of a population that would be recruited for a clinical trial of a potential AD-drug, such as from memory assessment clinics [66, 67]. For example, as observed in Supporting Information, S1 Table, in the dataset used in this study there is a high proportion of ApoE ε4 carriers, which is a significant genetic risk factor of AD. Thus, these individuals are at higher risk of developing the disease, creating significant selection bias issues. In addition, in the model we assumed long preclinical and prodromal AD stages and ADNI dataset is not sufficient to support this silent ‘incubation period’. Due to the nature of the data, we also believe that the real effect of hypothetical preventative treatments, as assessed in the current study, is underestimated, as CN individuals in ADNI move faster to the MCI state than expected in the general population. However, all this should not limit the applicability of the model and the major outputs of this work which illustrate the usefulness of clinical trial simulations in AD therapy area, and how the high variability in measurements could mask the true efficacy of potential AD treatments.

Despite the nature of AD and problems with the available data, the Markov model is a powerful tool for predicting the development and progression of the disease and the assessment of the impact of potential treatments in clinical trials. Ideally, the model described in this paper should be extended so that there are more disease states, given there is enough information for the derivation of reliable estimates of the model parameters. The model should also be extended for the estimation of covariate effects of risk factors on transitions among states and concomitantly on disease progression. Such covariates could include age, genetic background (for example presence/absence of the apolipoprotein ε4 (APOE ε4) genotype), gender and education, all of which are important risk factors for AD. Other factors, such as family history, capability, lifestyle (e.g. alcohol consumption), social status, health and environment can also be influential [68]. Adding such heterogeneity will be described in a subsequent publication. It would also be interesting to incorporate into the model changes in cognitive markers and biomarkers, such as beta-amyloid and total-tau [69], as well as MRI markers, even though the precise estimates of the transition probabilities over time, conditional on the levels of these markers, might be difficult using the current data.

Clinical trials are difficult to conduct and very expensive if carried out in large samples of patients observed over many years. Modelling and simulating AD trials are relatively low cost activities and they can be a powerful tool for improving the design of the actual clinical trials and increase the chances of the accurate assessment of treatment efficacy [70]. Improving the quality of the data is clearly essential for the determination of the importance of variance in clinical trials. The reduction of the variance in currently employed measures (whether biomarkers, brain scans or cognitive scores) will improve trial design, facilitate detecting a signal in the trial, shorten the trial times and reduce the number of patients enrolled. Ideal datasets should include frequent sampling and repeated assessments for the same individual [71]. Utilising the growing number of available AD datasets from many different countries, and performing systematic and standardised analyses, it should be possible to provide substantial information for improving clinical trial design [72]. However, extra caution will be needed when working with the different datasets. Among the available studies there is a variation in the diagnostic tools, in the markers measured and measurement methods used. There is also a little agreement on the definition of the disease states and selection criteria as well as the size and demographic characteristics of the samples. Pooling and standardisation of clinical trial data, as well as standardisation of the methods employed in future studies, will facilitate better prediction of disease course and allow for better interpretation of model predictions. It will help in the identification of the sources of variance, and consequently it will facilitate progress towards the precise assessment of new prophylactic and therapeutic treatments of AD.

## Supporting information

S1 Table Characteristics at baseline of the ADNI dataset that has been used in the current study. (DOCX)

S2 Table Number of transitions between the states of the Markov model at each time step in ADNI dataset. (DOCX)

S3 Table Expected proportion of AD cases (AD) at the end of the trial under different intervention scenarios in the case where at the beginning of the trial all individuals are at the CN state. Credible intervals (CI) and standard deviations (SD) are presented. The treatment is effective from the beginning of the trial, unless otherwise stated. The population size in each group is *N* = 1000.
(DOCX)

S4 Table Expected proportion of AD cases (AD) at the end of the trial under different intervention scenarios in the case where at the beginning of the trial all individuals are at the MCI state. Credible intervals (CI) and standard deviations (SD) are presented. The treatment is effective from the beginning of the trial, unless otherwise stated. The population size in each group is *N* = 1000.
(DOCX)

S5 Table *p*-values of the hypothesis tests for the equality of the expected proportions of AD cases in the untreated and treated groups under the different intervention scenarios in the case where at the beginning of the trial all individuals are at the CN state. The treatment is effective from the beginning of the trial, unless otherwise stated. The population size in each group is *N* = 1000.
(DOCX)

S6 Table *p*-values of the hypothesis tests for the equality of the expected proportions of AD cases in the untreated and treated groups under the different intervention scenarios in the case where at the beginning of the trial all individuals are at the MCI state. The treatment is effective from the beginning of the trial, unless otherwise stated. The population size in each group is *N* = 1000.
(DOCX)

S1 Fig Output of stochastic simulations: the expected proportion of AD cases over time and the effect of treatments that reduce the transition probability *p*_*CN*,*MCI*_ in a sample of CN individuals. The expected proportion of AD cases over time in the untreated group is presented by the solid line. The expected proportions after the administration of potential treatments that reduce the transition probability *p*_*CN*,*MCI*_ by a proportion *E*_*CN*,*MCI*_ are presented by the dashed lines. (A) *E*_*CN*,*MCI*_ = 0.2, (B) *E*_*CN*,*MCI*_ = 0.4, (C) *E*_*CN*,*MCI*_ = 0.6, (D) *E*_*CN*,*MCI*_ = 0.8. The value of *E* can be interpreted as the efficacy of the candidate treatment, with *E* = 0 implying no efficacy and *E* = 1 a 100% efficacy which stops progression. The ‘shaded area’ represents the 95% credible interval of the distribution of the proportion of AD cases. At the beginning of the trial all individuals are at the CN state. The population size in each group is *N* = 1000.
(DOCX)

S2 Fig Output of stochastic simulations: The expected proportion of AD cases over time and the effect of treatments that reduce the transition probabilities *p*_*CN*,*MCI*_ and *p*_*MCI*,*AD*_ in a sample of MCI individuals. The expected proportion of AD cases over time in the untreated group is presented by the solid line. The expected proportions after the administration of potential treatments that reduce the transition probabilities *p*_*CN*,*MCI*_ and *p*_*MCI*,*AD*_ by a proportion *E*_*CN*,*MCI*_ and *E*_*MCI*,*AD*_, respectively, are presented by the dashed lines. (A) *E*_*CN*,*MCI*_ = 0.2, *E*_*MCI*,*AD*_ = 0.2, (B) *E*_*CN*,*MCI*_ = 0.4, *E*_*MCI*,*AD*_ = 0.4, (C) *E*_*CN*,*MCI*_ = 0.6, *E*_*MCI*,*AD*_ = 0.6, (D) *E*_*CN*,*MCI*_ = 0.8, *E*_*MCI*,*AD*_ = 0.8. The value of *E* can be interpreted as the efficacy of the candidate treatment, with *E* = 0 implying no efficacy and *E* = 1 a 100% efficacy which stops progression. The ‘shaded area’ represents the 95% credible interval of the distribution of the proportion of AD cases. At the beginning of the trial all individuals are at the MCI state. The population size in each group is *N* = 1000.
(DOCX)

S3 Fig The expected proportion of AD cases at the end of a 10-year trial and the impact of time delays in the activation of various treatments in a sample of CN individuals. Treatments that reduce the transition probabilities (A) *p*_*CN*,*MCI*_ only, (B) *p*_*MCI*,*AD*_ only, (C) *p*_*CN*,*MCI*_ and *p*_*MCI*,*AD*_, by a proportion 0.5 are effective after some time delay. Circles represent the expected proportion of AD cases at the end of a 10-year trial and the error bars the 95% credible interval. At the beginning of the trial all individuals are at the CN state. The population size in each group is *N* = 1000.
(DOCX)

S4 Fig The expected proportion of AD cases at the end of a 5-year trial and the impact of time delays in the activation of a treatment in a sample of MCI individuals. The treatment that reduces the transition probabilities *p*_*CN*,*MCI*_ and *p*_*MCI*,*AD*_ by a proportion 0.5 is effective after some time delay. Circles represent the expected proportion of AD cases at the end of a 5-year trial and the error bars the 95% credible interval. At the beginning of the trial all individuals are at the MCI state. The population size in each group is *N* = 1000.
(DOCX)
