# Supplementary material for: The development of a stochastic mathematical model of Alzheimer’s disease to help improve the design of clinical trials of potential treatments
Source: PLoS One. 2018 Jan 29;13(1):e0190615. doi: 10.1371/journal.pone.0190615 (PMC5788351; doi:10.1371/journal.pone.0190615)
Supplement: S1 Table — (DOCX) [file pone.0190615.s001.docx]

**Table S1. Characteristics at baseline of the ADNI dataset that has been used in the current study.**

| **Total number of individuals considered** | | 1624 | | |
| --- | --- | --- | --- | --- |
| **Cognitive state at baseline** | | **CN** | **MCI** | **AD** |
| **Total number of individuals at each state** | | 417 | 866 | 341 |
| **Years of education** | $\boldsymbol{\leq}$ **12** | 45 | 147 | 83 |
|  | $\boldsymbol{>}$ **12** | 372 | 719 | 258 |
| **Gender** | **Women** | 208 | 354 | 152 |
|  | **Men** | 209 | 512 | 189 |
| **Genetic Background**  **(ApoE ε4)^*^** | **non-carriers** | 302 | 428 | 114 |
|  | **Carriers of 1 ε4 allele** | 103 | 341 | 160 |
|  | **Carriers of 2 ε4 alleles** | 11 | 94 | 65 |
| **Chronological age at baseline** | **≤70** | 73 | 294 | 81 |
|  | **70<Age≤80** | 269 | 399 | 167 |
|  | **>80** | 75 | 173 | 93 |
|  | **Average age** | 74.73 | 73.05 | 74.98 |
| ^*^In ADNI, this information is available only for 1618 out of the 1624 individuals considered. | | | | |
