# Supplementary material for: The development of a stochastic mathematical model of Alzheimer’s disease to help improve the design of clinical trials of potential treatments
Source: PLoS One. 2018 Jan 29;13(1):e0190615. doi: 10.1371/journal.pone.0190615 (PMC5788351; doi:10.1371/journal.pone.0190615)
Supplement: S2 Table — (DOCX) [file pone.0190615.s002.docx]

**Table S2.** **Number of transitions between the states of the Markov model at each time step in ADNI dataset.**

| **TO**  **FROM** | | **CN** | **MCI** | **AD** | **Withdrawal** |
| --- | --- | --- | --- | --- | --- |
|  |  | $\boldsymbol{t=1}$ **yr** | | | |
| $\boldsymbol{t=0}$ | **CN** = **417** | 375 | 12 | 0 | 23 |
|  | **MCI** = **866** | 25 | 662 | 104 | 69 |
|  | **AD** = **341** | 0 | 2 | 268 | 55 |
|  |  | $\boldsymbol{t=2}$ **yrs** | | | |
| $\boldsymbol{t=1}$ **yr** | **CN = 400** | 355 | 16 | 0 | 8 |
|  | **MCI = 676** | 17 | 462 | 105 | 51 |
|  | **AD = 372** | 0 | 4 | 241 | 95 |
|  |  | $\boldsymbol{t=3}$ **yrs** | | | |
| $\boldsymbol{t=2}$ **yrs** | **CN = 376** | 202 | 16 | 2 | 21 |
|  | **MCI = 486** | 13 | 342 | 45 | 36 |
|  | **AD = 351** | 0 | 4 | 145 | 90 |
|  |  | $\boldsymbol{t=4}$ **yrs** | | | |
| $\boldsymbol{t=3}$ **yrs** | **CN = 228** | 123 | 12 | 0 | 23 |
|  | **MCI = 379** | 10 | 215 | 29 | 26 |
|  | **AD = 197** | 0 | 4 | 99 | 42 |
|  |  | $\boldsymbol{t=5}$ **yrs** | | | |
| $\boldsymbol{t=4}$ **yrs** | **CN = 219** | 92 | 7 | 0 | 8 |
|  | **MCI = 249** | 4 | 96 | 5 | 11 |
|  | **AD = 134** | 0 | 2 | 61 | 16 |
|  |  | $\boldsymbol{t=6}$ **yrs** | | | |
| $\boldsymbol{t=5}$ **yrs** | **CN = 109** | 84 | 6 | 0 | 3 |
|  | **MCI = 120** | 1 | 48 | 9 | 1 |
|  | **AD = 75** | 0 | 1 | 47 | 11 |
|  |  | $\boldsymbol{t=7}$ **yrs** | | | |
| $\boldsymbol{t=6}$ **yrs** | **CN = 96** | 69 | 12 | 1 | 4 |
|  | **MCI = 71** | 0 | 51 | 4 | 7 |
|  | **AD = 61** | 0 | 0 | 34 | 13 |
|  |  | $\boldsymbol{t=8}$ **yrs** | | | |
| $\boldsymbol{t=7}$ **yrs** | **CN = 72** | 43 | 4 | 0 | 1 |
|  | **MCI = 68** | 4 | 41 | 12 | 4 |
|  | **AD = 40** | 0 | 0 | 21 | 10 |
|  |  | $\boldsymbol{t=9}$ **yrs** | | | |
| $\boldsymbol{t=8}$ **yrs** | **CN = 50** | 15 | 0 | 0 | 1 |
|  | **MCI = 48** | 0 | 29 | 6 | 1 |
|  | **AD = 34** | 0 | 0 | 24 | 4 |
|  |  | $\boldsymbol{t=10}$ **yrs** | | | |
| $\boldsymbol{t=9}$ **yrs** | **CN = 29** | 5 | 0 | 0 | 0 |
|  | **MCI = 35** | 1 | 12 | 2 | 0 |
|  | **AD = 31** | 0 | 0 | 9 | 1 |
