# Supplementary material for: The development of a stochastic mathematical model of Alzheimer’s disease to help improve the design of clinical trials of potential treatments
Source: PLoS One. 2018 Jan 29;13(1):e0190615. doi: 10.1371/journal.pone.0190615 (PMC5788351; doi:10.1371/journal.pone.0190615)
Supplement: S3 Table — Credible intervals (CI) and standard deviations (SD) are presented. The treatment is effective from the beginning of the trial, unless otherwise stated. The population size in each group is N = 1000. (DOCX) [file pone.0190615.s003.docx]

**Table S3.** **Expected proportion of AD cases (AD) at the end of the trial under different intervention scenarios in the case where at the beginning of the trial all individuals are at the CN state.** Credible intervals (CI) and standard deviations (SD) are presented. The treatment is effective from the beginning of the trial, unless otherwise stated. The population size in each group is $N=1000$.

|  | **At the end of a**  **5-year trial** | **At the end of a**  **10-year trial** |
| --- | --- | --- |
| **No Intervention** | AD = 0.026  CI = (0.017, 0.036)  SD = 0.005 | AD = 0.051  CI = (0.038, 0.066)  SD = 0.007 |
| $\boldsymbol{E}_{\boldsymbol{CN,MCI}}$ **= 0.2** | AD = 0.021  CI = (0.013, 0.03)  SD = 0.004 | AD = 0.042  CI = (0.03, 0.055)  SD = 0.006 |
| $\boldsymbol{E}_{\boldsymbol{CN,MCI}}$ **= 0.4** | AD = 0.016  CI = (0.009, 0.024)  SD = 0.004 | AD = 0.033  CI = (0.022, 0.044)  SD = 0.006 |
| $\boldsymbol{E}_{\boldsymbol{CN,MCI}}$ **= 0.5** | AD = 0.014  CI = (0.007, 0.021)  SD = 0.004 | AD = 0.028  CI = (0.018, 0.038)  SD = 0.005 |
| $\boldsymbol{E}_{\boldsymbol{CN,MCI}}$ **= 0.6** | AD = 0.011  CI = (0.005, 0.018)  SD = 0.003 | AD = 0.023  CI = (0.014, 0.032)  SD = 0.005 |
| $\boldsymbol{E}_{\boldsymbol{CN,MCI}}$ **= 0.8** | AD = 0.006  CI = (0.001, 0.011)  SD = 0.002 | AD = 0.012  CI = (0.006, 0.019)  SD = 0.003 |
| $\boldsymbol{E}_{\boldsymbol{MCI,AD}}$ **= 0.2** | AD = 0.022  CI = (0.013, 0.031)  SD = 0.005 | AD = 0.044  CI = (0.031, 0.057)  SD = 0.006 |
| $\boldsymbol{E}_{\boldsymbol{MCI,AD}}$ **= 0.4** | AD = 0.017  CI = (0.009, 0.025)  SD = 0.004 | AD = 0.035  CI = (0.024, 0.047)  SD = 0.006 |
| $\boldsymbol{E}_{\boldsymbol{MCI,AD}}$ **= 0.5** | AD = 0.014  CI = (0.008, 0.022)  SD = 0.004 | AD = 0.030  CI = (0.02, 0.041)  SD = 0.005 |
| $\boldsymbol{E}_{\boldsymbol{MCI,AD}}$ **= 0.6** | AD = 0.012  CI = (0.006, 0.019)  SD = 0.003 | AD = 0.025  CI = (0.016, 0.035)  SD = 0.005 |
| $\boldsymbol{E}_{\boldsymbol{MCI,AD}}$ **= 0.8** | AD = 0.006  CI = (0.002, 0.011)  SD = 0.002 | AD = 0.013  CI = (0.007, 0.021)  SD = 0.004 |
| $\boldsymbol{E}_{\boldsymbol{CN,MCI}}$ **= 0.2,** $\boldsymbol{E}_{\boldsymbol{MCI,AD}}$ **= 0.2** | AD = 0.018  CI = (0.01, 0.026)  SD = 0.004 | AD = 0.036  CI = (0.025, 0.048)  SD = 0.006 |
| $\boldsymbol{E}_{\boldsymbol{CN,MCI}}$ **= 0.4,** $\boldsymbol{E}_{\boldsymbol{MCI,AD}}$ **= 0.4** | AD = 0.010  CI = (0.005, 0.017)  SD = 0.003 | AD = 0.022  CI = (0.014, 0.032)  SD = 0.005 |
| $\boldsymbol{E}_{\boldsymbol{CN,MCI}}$ **= 0.5,** $\boldsymbol{E}_{\boldsymbol{MCI,AD}}$ **= 0.5** | AD = 0.007  CI = (0.003, 0.013)  SD = 0.003 | AD = 0.016  CI = (0.009, 0.025)  SD = 0.004 |
| $\boldsymbol{E}_{\boldsymbol{CN,MCI}}$ **= 0.6,** $\boldsymbol{E}_{\boldsymbol{MCI,AD}}$ **= 0.6** | AD = 0.005  CI = (0.001, 0.009)  SD = 0.002 | AD = 0.011  CI = (0.005, 0.018)  SD = 0.003 |
| $\boldsymbol{E}_{\boldsymbol{CN,MCI}}$ **= 0.8,** $\boldsymbol{E}_{\boldsymbol{MCI,AD}}$ **= 0.8** | AD = 0.001  CI = (0, 0.004)  SD = 0.001 | AD = 0.003  CI = (0, 0.007)  SD = 0.002 |
| $\boldsymbol{E}_{\boldsymbol{CN,MCI}}$ **= 0.5,**  **1yr delay** | AD = 0.017  CI = (0.01, 0.026)  SD = 0.004 | AD = 0.031  CI = (0.02, 0.042)  SD = 0.005 |
| $\boldsymbol{E}_{\boldsymbol{CN,MCI}}$ **= 0.5,**  **2yr delay** | AD = 0.021  CI = (0.012, 0.03)  SD = 0.004 | AD = 0.034  CI = (0.023, 0.045)  SD = 0.006 |
| $\boldsymbol{E}_{\boldsymbol{CN,MCI}}$ **= 0.5,**  **3yr delay** | AD = 0.023  CI = (0.015, 0.033)  SD = 0.005 | AD = 0.037  CI = (0.025, 0.049)  SD = 0.006 |
| $\boldsymbol{E}_{\boldsymbol{CN,MCI}}$ **= 0.5,**  **4yr delay** | AD = 0.025  CI = (0.016, 0.0355)  SD = 0.005 | AD = 0.039  CI = (0.028, 0.052)  SD = 0.006 |
| $\boldsymbol{E}_{\boldsymbol{CN,MCI}}$ **= 0.5,**  **5yr delay** |  | AD = 0.042  CI = (0.03, 0.055)  SD = 0.006 |
| $\boldsymbol{E}_{\boldsymbol{CN,MCI}}$ **= 0.5,**  **6yr delay** |  | AD = 0.045  CI = (0.033, 0.058)  SD = 0.006 |
| $\boldsymbol{E}_{\boldsymbol{MCI,AD}}$ **= 0.5,**  **1yr delay** | AD = 0.014  CI = (0.008, 0.022)  SD = 0.004 | AD = 0.030  CI = (0.02, 0.041)  SD = 0.005 |
| $\boldsymbol{E}_{\boldsymbol{MCI,AD}}$ **= 0.5,**  **2yr delay** | AD = 0.015  CI = (0.008, 0.024)  SD = 0.004 | AD = 0.030  CI = (0.02, 0.041)  SD = 0.005 |
| $\boldsymbol{E}_{\boldsymbol{MCI,AD}}$ **= 0.5,**  **3yr delay** | AD = 0.018  CI = (0.01, 0.026)  SD = 0.004 | AD = 0.031  CI = (0.02, 0.042)  SD = 0.005 |
| $\boldsymbol{E}_{\boldsymbol{MCI,AD}}$ **= 0.5,**  **4yr delay** | AD = 0.021  CI = (0.013, 0.03)  SD = 0.004 | AD = 0.031  CI = (0.021, 0.043)  SD = 0.006 |
| $\boldsymbol{E}_{\boldsymbol{MCI,AD}}$ **= 0.5,**  **5yr delay** |  | AD = 0.033  CI = (0.022, 0.045)  SD = 0.006 |
| $\boldsymbol{E}_{\boldsymbol{MCI,AD}}$ **= 0.5,**  **6yr delay** |  | AD = 0.035  CI = (0.024, 0.046)  SD = 0.006 |
| $\boldsymbol{E}_{\boldsymbol{CN,MCI}}$ **= 0.5,** $\boldsymbol{E}_{\boldsymbol{MCI,AD}}$ **= 0.5,**  **1yr delay** | AD = 0.010  CI = (0.004, 0.016)  SD = 0.003 | AD = 0.018  CI = (0.01, 0.027)  SD = 0.004 |
| $\boldsymbol{E}_{\boldsymbol{CN,MCI}}$ **= 0.5,** $\boldsymbol{E}_{\boldsymbol{MCI,AD}}$ **= 0.5,**  **2yr delay** | AD = 0.013  CI = (0.006, 0.02)  SD = 0.003 | AD = 0.020  CI = (0.012, 0.03)  SD = 0.004 |
| $\boldsymbol{E}_{\boldsymbol{CN,MCI}}$ **= 0.5,** $\boldsymbol{E}_{\boldsymbol{MCI,AD}}$ **= 0.5,**  **3yr delay** | AD = 0.016  CI = (0.009, 0.024)  SD = 0.004 | AD = 0.023  CI = (0.014, 0.032)  SD = 0.005 |
| $\boldsymbol{E}_{\boldsymbol{CN,MCI}}$ **= 0.5,** $\boldsymbol{E}_{\boldsymbol{MCI,AD}}$ **= 0.5,**  **4yr delay** | AD = 0.020  CI = (0.012, 0.03)  SD = 0.004 | AD = 0.025  CI = (0.016, 0.035)  SD = 0.005 |
| $\boldsymbol{E}_{\boldsymbol{CN,MCI}}$ **= 0.5,** $\boldsymbol{E}_{\boldsymbol{MCI,AD}}$ **= 0.5,**  **5yr delay** |  | AD = 0.028  CI = (0.018, 0.039)  SD = 0.005 |
| $\boldsymbol{E}_{\boldsymbol{CN,MCI}}$ **= 0.5,** $\boldsymbol{E}_{\boldsymbol{MCI,AD}}$ **= 0.5,**  **6yr delay** |  | AD = 0.031  CI = (0.021, 0.043)  SD = 0.005 |
