# Supplementary material for: The development of a stochastic mathematical model of Alzheimer’s disease to help improve the design of clinical trials of potential treatments
Source: PLoS One. 2018 Jan 29;13(1):e0190615. doi: 10.1371/journal.pone.0190615 (PMC5788351; doi:10.1371/journal.pone.0190615)
Supplement: S4 Table — Credible intervals (CI) and standard deviations (SD) are presented. The treatment is effective from the beginning of the trial, unless otherwise stated. The population size in each group is N = 1000. (DOCX) [file pone.0190615.s004.docx]

**Table S4. Expected proportion of AD cases (AD) at the end of the trial under different intervention scenarios in the case where at the beginning of the trial all individuals are at the MCI state.** Credible intervals (CI) and standard deviations (SD) are presented. The treatment is effective from the beginning of the trial, unless otherwise stated. The population size in each group is $N=1000$.

|  | **At the end of a**  **5-year trial** |
| --- | --- |
| **No Intervention** | AD = 0.201  CI = (0.176, 0.226)  SD = 0.013 |
| $\boldsymbol{E}_{\boldsymbol{CN,MCI}}$ **= 0.2,** $\boldsymbol{E}_{\boldsymbol{MCI,AD}}$ **= 0.2** | AD = 0.169  CI = (0.146, 0.192)  SD = 0.012 |
| $\boldsymbol{E}_{\boldsymbol{CN,MCI}}$ **= 0.4,** $\boldsymbol{E}_{\boldsymbol{MCI,AD}}$ **= 0.4** | AD = 0.133  CI = (0.113, 0.155)  SD = 0.011 |
| $\boldsymbol{E}_{\boldsymbol{CN,MCI}}$ **= 0.5,** $\boldsymbol{E}_{\boldsymbol{MCI,AD}}$ **= 0.5** | AD = 0.114  CI = (0.094, 0.134)  SD = 0.010 |
| $\boldsymbol{E}_{\boldsymbol{CN,MCI}}$ **= 0.6,** $\boldsymbol{E}_{\boldsymbol{MCI,AD}}$ **= 0.6** | AD = 0.094  CI = (0.076, 0.112)  SD = 0.009 |
| $\boldsymbol{E}_{\boldsymbol{CN,MCI}}$ **= 0.8,** $\boldsymbol{E}_{\boldsymbol{MCI,AD}}$ **= 0.8** | AD = 0.049  CI = (0.036, 0.063)  SD = 0.007 |
| $\boldsymbol{E}_{\boldsymbol{CN,MCI}}$ **= 0.5,** $\boldsymbol{E}_{\boldsymbol{MCI,AD}}$ **= 0.5,**  **1yr delay** | AD = 0.127  CI = (0.107, 0.148)  SD = 0.011 |
| $\boldsymbol{E}_{\boldsymbol{CN,MCI}}$ **= 0.5,** $\boldsymbol{E}_{\boldsymbol{MCI,AD}}$ **= 0.5,**  **2yr delay** | AD = 0.143  CI = (0.121, 0.165)  SD = 0.011 |
| $\boldsymbol{E}_{\boldsymbol{CN,MCI}}$ **= 0.5,** $\boldsymbol{E}_{\boldsymbol{MCI,AD}}$ **= 0.5,**  **3yr delay** | AD = 0.160  CI = (0.137, 0.183)  SD = 0.012 |
| $\boldsymbol{E}_{\boldsymbol{CN,MCI}}$ **= 0.5,** $\boldsymbol{E}_{\boldsymbol{MCI,AD}}$ **= 0.5,**  **4yr delay** | AD = 0.179  CI = (0.156, 0.203)  SD = 0.012 |
