# Supplementary material for: The development of a stochastic mathematical model of Alzheimer’s disease to help improve the design of clinical trials of potential treatments
Source: PLoS One. 2018 Jan 29;13(1):e0190615. doi: 10.1371/journal.pone.0190615 (PMC5788351; doi:10.1371/journal.pone.0190615)
Supplement: S5 Table — The treatment is effective from the beginning of the trial, unless otherwise stated. The population size in each group is N = 1000. (DOCX) [file pone.0190615.s005.docx]

**Table S5.** $\boldsymbol{p}$**-values of the hypothesis tests for the equality of the expected proportions of AD cases in the untreated and treated groups under the different intervention scenarios in the case where at the beginning of the trial all individuals are at the CN state.** The treatment is effective from the beginning of the trial, unless otherwise stated. The population size in each group is $N=1000$.

|  | | $\boldsymbol{p}$**-values** | |
| --- | --- | --- | --- |
|  |  | **Comparison with the untreated group at the end of a**  **5-year trial** | **Comparison with the untreated group at the end of a**  **10-year trial** |
| **Intervention** | $\boldsymbol{E}_{\boldsymbol{CN,MCI}}$ **= 0.2** | 0.4710 | 0.3432 |
|  | $\boldsymbol{E}_{\boldsymbol{CN,MCI}}$ **= 0.4** | 0.1253 | **0.0409** |
|  | $\boldsymbol{E}_{\boldsymbol{CN,MCI}}$ **= 0.5** | **0.0447** | **0.0074** |
|  | $\boldsymbol{E}_{\boldsymbol{CN,MCI}}$ **= 0.6** | **0.0120** | **7.2167E-04** |
|  | $\boldsymbol{E}_{\boldsymbol{CN,MCI}}$ **= 0.8** | **2.2985E-04** | **4.2629E-07** |
|  | $\boldsymbol{E}_{\boldsymbol{MCI,AD}}$ **= 0.2** | 0.5170 | 0.4250 |
|  | $\boldsymbol{E}_{\boldsymbol{MCI,AD}}$ **= 0.4** | 0.1472 | 0.0726 |
|  | $\boldsymbol{E}_{\boldsymbol{MCI,AD}}$ **= 0.5** | 0.0591 | **0.0167** |
|  | $\boldsymbol{E}_{\boldsymbol{MCI,AD}}$ **= 0.6** | **0.0167** | **0.0021** |
|  | $\boldsymbol{E}_{\boldsymbol{MCI,AD}}$ **= 0.8** | **3.2992E-04** | **1.6764E-06** |
|  | $\boldsymbol{E}_{\boldsymbol{CN,MCI}}$ **= 0.2,** $\boldsymbol{E}_{\boldsymbol{MCI,AD}}$ **= 0.2** | 0.1906 | 0.0980 |
|  | $\boldsymbol{E}_{\boldsymbol{CN,MCI}}$ **= 0.4,** $\boldsymbol{E}_{\boldsymbol{MCI,AD}}$ **= 0.4** | **0.0085** | **5.8180E-04** |
|  | $\boldsymbol{E}_{\boldsymbol{CN,MCI}}$ **= 0.5,** $\boldsymbol{E}_{\boldsymbol{MCI,AD}}$ **= 0.5** | **0.0011** | **1.5272E-05** |
|  | $\boldsymbol{E}_{\boldsymbol{CN,MCI}}$ **= 0.6,** $\boldsymbol{E}_{\boldsymbol{MCI,AD}}$ **= 0.6** | **1.1473E-04** | **2.2422E-07** |
|  | $\boldsymbol{E}_{\boldsymbol{CN,MCI}}$ **= 0.8,** $\boldsymbol{E}_{\boldsymbol{MCI,AD}}$ **= 0.8** | **1.6806E-06** | **3.2274E-11** |
|  | $\boldsymbol{E}_{\boldsymbol{CN,MCI}}$ **= 0.5,**  **1yr delay** | 0.1694 | **0.0198** |
|  | $\boldsymbol{E}_{\boldsymbol{CN,MCI}}$ **= 0.5,**  **2yr delay** | 0.4240 | 0.0508 |
|  | $\boldsymbol{E}_{\boldsymbol{CN,MCI}}$ **= 0.5,**  **3yr delay** | 0.7044 | 0.1139 |
|  | $\boldsymbol{E}_{\boldsymbol{CN,MCI}}$ **= 0.5,**  **4yr delay** | 0.9195 | 0.2091 |
|  | $\boldsymbol{E}_{\boldsymbol{CN,MCI}}$ **= 0.5,**  **5yr delay** | 1.0117 | 0.3568 |
|  | $\boldsymbol{E}_{\boldsymbol{CN,MCI}}$ **= 0.5,**  **6yr delay** | 1.0083 | 0.5203 |
|  | $\boldsymbol{E}_{\boldsymbol{MCI,AD}}$ **= 0.5,**  **1yr delay** | 0.0640 | **0.0174** |
|  | $\boldsymbol{E}_{\boldsymbol{MCI,AD}}$ **= 0.5,**  **2yr delay** | 0.0949 | **0.0183** |
|  | $\boldsymbol{E}_{\boldsymbol{MCI,AD}}$ **= 0.5,**  **3yr delay** | 0.1885 | **0.0212** |
|  | $\boldsymbol{E}_{\boldsymbol{MCI,AD}}$ **= 0.5,**  **4yr delay** | 0.4285 | **0.0269** |
|  | $\boldsymbol{E}_{\boldsymbol{MCI,AD}}$ **= 0.5,**  **5yr delay** | 0.9565 | **0.0404** |
|  | $\boldsymbol{E}_{\boldsymbol{MCI,AD}}$ **= 0.5,**  **6yr delay** | 1.0020 | 0.0690 |
|  | $\boldsymbol{E}_{\boldsymbol{CN,MCI}}$ **= 0.5,** $\boldsymbol{E}_{\boldsymbol{MCI,AD}}$ **= 0.5,**  **1yr delay** | **0.0054** | **5.3067E-05** |
|  | $\boldsymbol{E}_{\boldsymbol{CN,MCI}}$ **= 0.5,** $\boldsymbol{E}_{\boldsymbol{MCI,AD}}$ **= 0.5,**  **2yr delay** | **0.0282** | **1.9223E-04** |
|  | $\boldsymbol{E}_{\boldsymbol{CN,MCI}}$ **= 0.5,** $\boldsymbol{E}_{\boldsymbol{MCI,AD}}$ **= 0.5,**  **3yr delay** | 0.1232 | **7.0886E-04** |
|  | $\boldsymbol{E}_{\boldsymbol{CN,MCI}}$ **= 0.5,** $\boldsymbol{E}_{\boldsymbol{MCI,AD}}$ **= 0.5,**  **4yr delay** | 0.4048 | **0.0024** |
|  | $\boldsymbol{E}_{\boldsymbol{CN,MCI}}$ **= 0.5,** $\boldsymbol{E}_{\boldsymbol{MCI,AD}}$ **= 0.5,**  **5yr delay** | 0.9637 | **0.0083** |
|  | $\boldsymbol{E}_{\boldsymbol{CN,MCI}}$ **= 0.5,** $\boldsymbol{E}_{\boldsymbol{MCI,AD}}$ **= 0.5,**  **6yr delay** | 1.0031 | **0.0263** |
