# Supplementary material for: The development of a stochastic mathematical model of Alzheimer’s disease to help improve the design of clinical trials of potential treatments
Source: PLoS One. 2018 Jan 29;13(1):e0190615. doi: 10.1371/journal.pone.0190615 (PMC5788351; doi:10.1371/journal.pone.0190615)
Supplement: S1 Fig — The expected proportion of AD cases over time in the untreated group is presented by the solid line. The expected proportions after the administration of potential treatments that reduce the transition probability pCN,MCI by a proportion ECN,MCI are presented by the dashed lines. (A) ECN,MCI = 0.2, (B) ECN,MCI = 0.4, (C) ECN,MCI = 0.6, (D) ECN,MCI = 0.8. The value of E can be interpreted as the efficacy of the candidate treatment, with E = 0 implying no efficacy and E = 1 a 100% efficacy which stops progression. The ‘shaded area’ represents the 95% credible interval of the distribution of the proportion of AD cases. At the beginning of the trial all individuals are at the CN state. The population size in each group is N = 1000. (DOCX) [file pone.0190615.s007.docx]

**
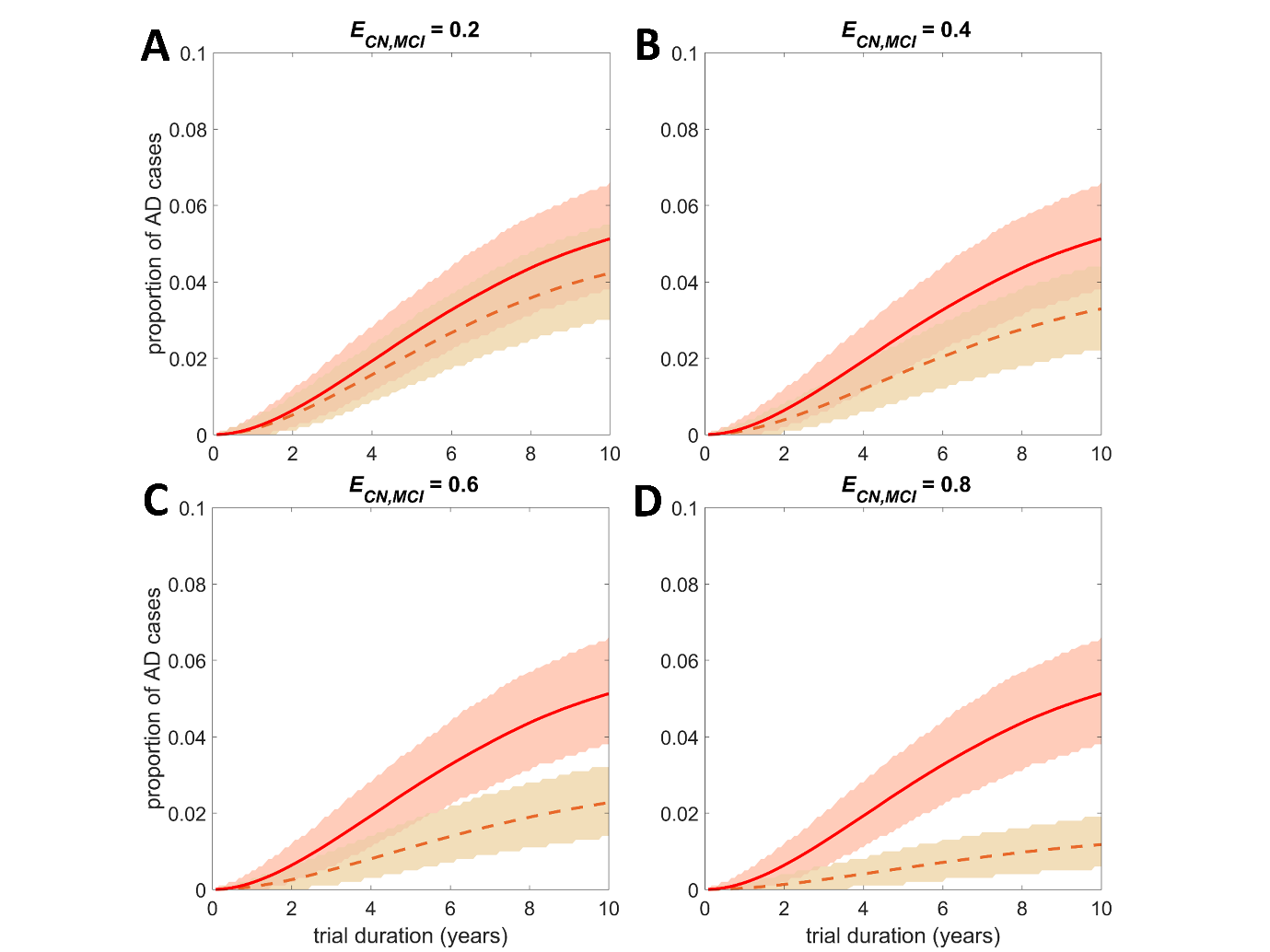
**

**Fig S1. Output of stochastic simulations: the expected proportion of AD cases over time and the effect of treatments that reduce the transition probability** $\boldsymbol{p}_{\boldsymbol{CN}\mathbf{,}\boldsymbol{MCI}}$ **in a sample of CN individuals.** The expected proportion of AD cases over time in the untreated group is presented by the solid line. The expected proportions after the administration of potential treatments that reduce the transition probability $p_{CN,MCI}$ by a proportion $E_{CN,MCI}$ are presented by the dashed lines. (A) $E_{CN,MCI}=0.2$, (B) $E_{CN,MCI}=0.4$, (C) $E_{CN,MCI}=0.6$, (D) $E_{CN,MCI}=0.8$. The value of $E$ can be interpreted as the efficacy of the candidate treatment, with $E=0$ implying no efficacy and $E=1$ a 100% efficacy which stops progression. The ‘shaded area’ represents the 95% credible interval of the distribution of the proportion of AD cases. At the beginning of the trial all individuals are at the CN state. The population size in each group is $N=1000$.
