# Supplementary material for: The development of a stochastic mathematical model of Alzheimer’s disease to help improve the design of clinical trials of potential treatments
Source: PLoS One. 2018 Jan 29;13(1):e0190615. doi: 10.1371/journal.pone.0190615 (PMC5788351; doi:10.1371/journal.pone.0190615)
Supplement: S3 Fig — Treatments that reduce the transition probabilities (A) pCN,MCI only, (B) pMCI,AD only, (C) pCN,MCI and pMCI,AD, by a proportion 0.5 are effective after some time delay. Circles represent the expected proportion of AD cases at the end of a 10-year trial and the error bars the 95% credible interval. At the beginning of the trial all individuals are at the CN state. The population size in each group is N = 1000. (DOCX) [file pone.0190615.s009.docx]

**
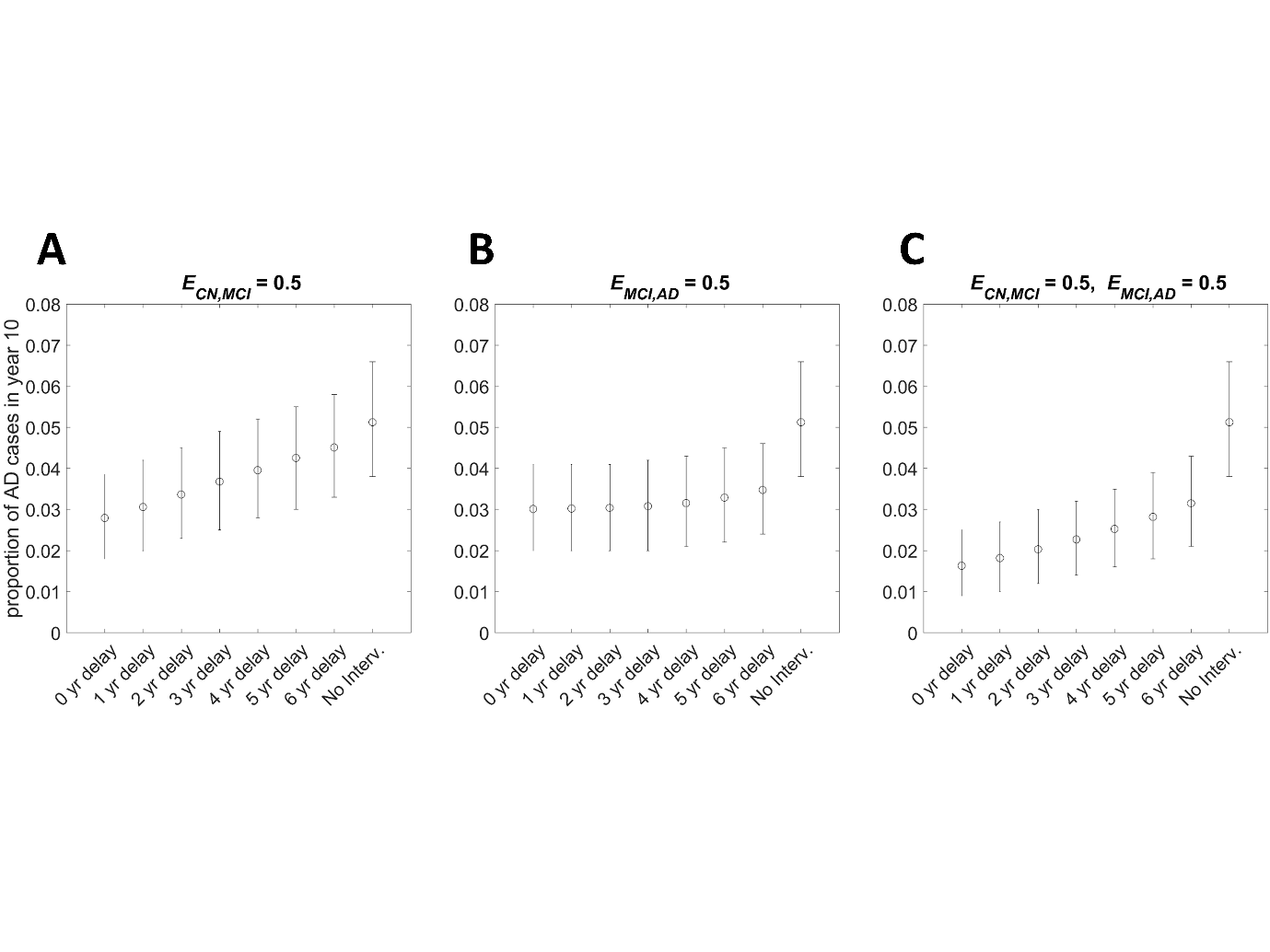
**

**Fig S3. The expected proportion of AD cases at the end of a 10-year trial and the impact of time delays in the activation of various treatments in a sample of CN individuals.** Treatments that reduce the transition probabilities (A) $p_{CN,MCI}$ only, (B) $p_{MCI,AD}$ only, (C) $p_{CN,MCI}$ and $p_{MCI,AD}$, by a proportion $0.5$ are effective after some time delay. Circles represent the expected proportion of AD cases at the end of a 10-year trial and the error bars the 95% credible interval. At the beginning of the trial all individuals are at the CN state. The population size in each group is $N=1000$.
