# Supplementary material for: The development of a stochastic mathematical model of Alzheimer’s disease to help improve the design of clinical trials of potential treatments
Source: PLoS One. 2018 Jan 29;13(1):e0190615. doi: 10.1371/journal.pone.0190615 (PMC5788351; doi:10.1371/journal.pone.0190615)
Supplement: S4 Fig — The treatment that reduces the transition probabilities pCN,MCI and pMCI,AD by a proportion 0.5 is effective after some time delay. Circles represent the expected proportion of AD cases at the end of a 5-year trial and the error bars the 95% credible interval. At the beginning of the trial all individuals are at the MCI state. The population size in each group is N = 1000. (DOCX) [file pone.0190615.s010.docx]

**
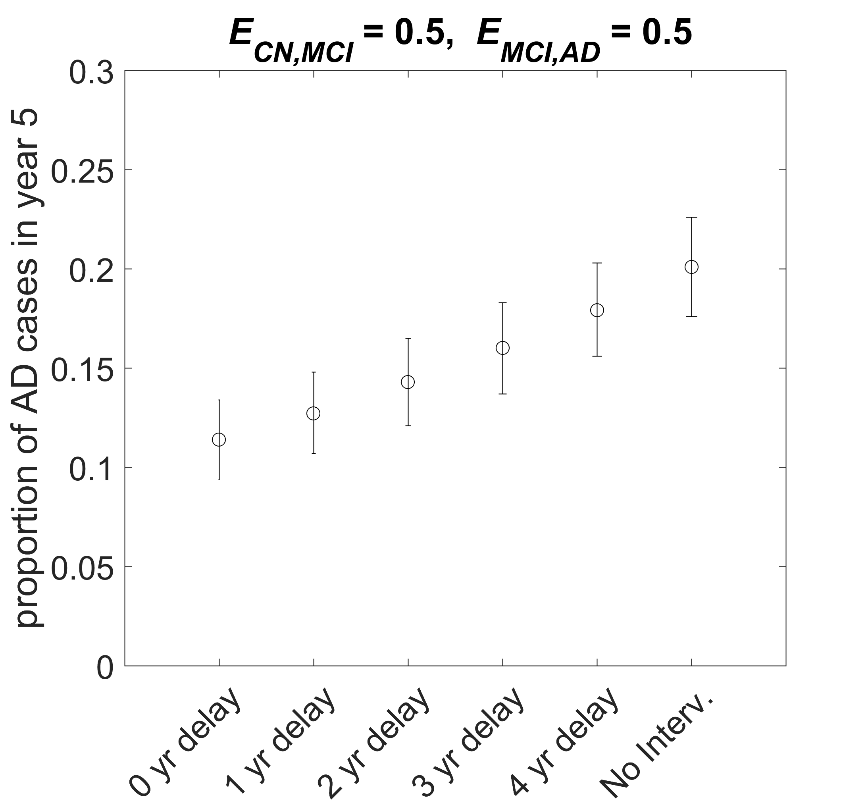
**

**Fig S4. The expected proportion of AD cases at the end of a 5-year trial and the impact of time delays in the activation of a treatment in a sample of MCI individuals.** The treatment that reduces the transition probabilities $p_{CN,MCI}$ and $p_{MCI,AD}$ by a proportion $0.5$ is effective after some time delay. Circles represent the expected proportion of AD cases at the end of a 5-year trial and the error bars the 95% credible interval. At the beginning of the trial all individuals are at the MCI state. The population size in each group is $N=1000$.
